# Does Organizational Cronyism Lead to Lower Employee Performance? Examining the Mediating Role of Employee Engagement and Moderating Role of Islamic Work Ethics

**DOI:** 10.3389/fpsyg.2020.579560

**Published:** 2020-10-02

**Authors:** Sadia Shaheen, Sehar Zulfiqar, Sharjeel Saleem, Gulshan Shehazadi

**Affiliations:** ^1^Lyallpur Business School, Government College University Faisalabad, Faisalabad, Pakistan; ^2^Department of Management Sciences, National University of Modern Languages, Islamabad, Pakistan

**Keywords:** organizational cronyism, Islamic work ethics, work engagement, employee performance, conservation of resources theory

## Abstract

In this research, using a time-lagged approach, we investigated the relationship between organizational cronyism and employee performance. Drawing on the conservation of resources theory, we tested the mediating role of employee engagement in the relationship between organizational cronyism and employee performance. We also examined how Islamic work ethics moderated the relationship between organizational cronyism and work engagement. The study, with a total of 267 participants, was conducted in the healthcare sector of Pakistan. The results revealed that organizational cronyism was negatively related to employee performance. The analyses confirmed the mediating role of work engagement in the relationship between organizational cronyism and employee performance. Similarly, Islamic work ethics moderated the relationship between organizational cronyism and work engagement. Implications for future research as well as managerial implications of our findings along with the limitations and future research directions are also discussed.

## Introduction

Organizational cronyism is defined as the bestowing of favors to friends, colleagues, and associates based on personal relationships and connections rather than actual performance standards (Khatri and Tsang, 2003; Turhan, 2014). In the presence of organizational cronyism, certain employees are favored, who are known as cronies and others are ignored who are known as non-cronies (Khatri and Tsang, 2003; Khatri et al., 2006; Khatri, 2017). According to the Conservation of Resource (COR) theory, it can be argued that organizational cronyism exhausts employees’ resources; employees who are ignored and unfavored at the workplace respond with less positive and more negative behaviors toward completing their tasks. Therefore, organizational cronyism can be considered as a workplace stressor (Khatri and Tsang, 2003; Turhan, 2014).

Employees expect fair and equitable treatment at all levels in all types of organizations. The available literature on the conception of organizational cronyism depicts that both public and private sectors are not free from the practices of organizational cronyism. Employees who are ignored have to bear the loss in financial and non-financial matters (Bernstein et al., 2010). Where public sector employees are not safe from such evil practices, private sector employees also have to encounter such exploitation of power by their leaders and managers (Shaheen et al., 2017). Practices of organizational cronyism have the potential to damage the goodwill of private sector organizations (Begley et al., 2010). Keles et al. (2011) found organizational cronyism, favoritism, and nepotism to be negatively related to trust in private family-owned firms.

When employee’s perception of fair treatment is violated, it might result in hostile consequences for individuals as well as for the organizations (Peng et al., 2016; van Gilst et al., 2020); for instance, it has the potential to decrease desired job outcomes (e.g., organizational commitment, job satisfaction, and organizational citizenship behavior) and increased undesirable job outcomes (e.g., deviant workplace behavior, turnover intention, negligent behavior, and adverse employee performance) (Arasli and Tumer, 2008; Büte, 2011; Keles et al., 2011; Turhan, 2014; Saleem et al., 2018; Daðli and Akyol, 2019). Building upon the COR perspective, the current study aims to examine the impact of organizational cronyism on employee performance and its underlying mechanism. A number of studies suggest that the best way to increase employee performance is to emphasize on boosting employee engagement (Rich et al., 2010). Therefore, our study would examine the impact of organizational cronyism through the novel lens of employee engagement.

Additionally, in contrast to the traditional approaches emphasizing only on the negative consequences of organizational cronyism, our study seeks to highlight the importance of contextual factors in understanding the effect of organizational cronyism on employee outcomes. Although modern literature highlighted several adverse outcomes of organizational cronyism, how the contextual factors affect the relationship between organizational cronyism and individual outcomes are underemphasized (Khatri, 2011; Turhan, 2014; Saleem et al., 2018). In the light of justice and organizational politics literature, it is suggested that individual reactions toward injustice and organizational cronyism may differ in different contextual settings and across cultures (Khatri, 2017).

Further, applying the COR theory perspective (Hobfoll, 2001), it can be argued that some contextual factors can buffer the relationship between workplace stressors and workplace outcomes. These factors may include religious assets that may serve as a coping mechanism for employees and avert them from demonstrating negative attitudes and behaviors even when they face organizational cronyism. Religion is defined as a “system of beliefs, practices, customs and ceremonies rooted in a culture; a view of the individual’s relationship to the universe; a moral and ethical code; and a community of adherents providing social relationships” (Shaw et al., 2005). Religion has always been considered as a persuader for employees to achieve their goals and objectives (Ali et al., 1995). Due to this reason, Islamic work ethics (IWE) may be utilized for motivating employees and strengthening their engagement, loyalty, and commitment to their jobs and tolerate discrimination and organizational cronyism at the workplace (Ali and Gibbs, 1998; Aryee et al., 2002). IWE is defined as an ethical set of standards that distinguish between good and bad or right and wrong in fulfilling the religious and social obligations (Beekun, 2019). IWE is based on the teachings of the Quran (The Holy book of Muslims) and Hadiths (The sayings of Prophet Muhammad (Peace Be upon him-PBUH).

Several researchers suggest examining the moderating role of IWE in the relationship between workplace stressors (e.g., organizational cronyism) and work outcomes (e.g., work engagement). We argue that reaction toward organizational cronyism is relatively low, where employees give preference to IWE and adopt the practices of IWE (Ali and Al-Kazemi, 2007; Marri, 2012; Khan et al., 2013). The teachings of Islam educate the Muslims that there is no retaliation in Islam; instead, clemency is strongly appreciated and valued in Islam (Ali and Al-Kazemi, 2007; Ali and Al-Owaihan, 2008). In the presence of high IWE, employees are motivated to be patient with the mistreatment extended by others, such as organizational cronyism and remain honest with their duties. With this faith that each act will be observed by Allah, an employee who is with high IWE performs his/her duties with full sincerity and engagement (Marri, 2012).

The current study will prove fruitful in understanding the implications of the COR theory (Hobfoll, 2001) in Eastern settings, which has been previously developed and tested predominantly in Western settings. Organizational researchers also suggest to examine and extend organizational theories in Eastern settings for the sake of generalizability (Tsui et al., 2007). Hence, the current study helps in filling this gap. The buffering role of IWE is proposed in our study that can help to mitigate the detrimental consequences of organizational cronyism on employee outcomes. It is extremely relevant due to the increasing workplace diversity and globalization that has compelled researchers to examine the religious as well as social factors affecting employee attitudes and behaviors (Eastman and Santoro, 2003). Thus, this study helps in filling the gap by understanding the Muslim population’s behaviors and attitudes that is increasing 1.5 times quicker than the rest of the world and is currently comprised of 23.5 percent of the entire population (Johnson et al., 2013).

Accordingly, the primary objective of this study is to broaden the literature on organizational cronyism by uncovering its impact on imperative job outcomes (i.e., employee performance) via the mechanism of employee engagement. Second, our study, using the resource perspective, tests the interplay between organizational cronyism and IWE in predicting work engagement. Our study contributes to the literature in the following ways: First, the current study draws on the COR theory (Hobfoll, 2001) to investigate the impact of organizational cronyism on employee performance via the mechanism of employee engagement. Second, the buffering role of IWE is proposed in the relationship between organizational cronyism and work engagement. Thus, we add a new resource IWE in the COR theory that is expected to mitigate the negative impact of organizational cronyism on work engagement. Third, this study attempts to shed light on the practices of organizational cronyism in the context of Asian culture where these evil practices are damaging the well-being of the individuals as well as organizations but are yet less explored and underemphasized by academic researchers.

## Theory and Hypotheses

By considering organizational cronyism as workplace stressor, the current model is based on the COR theory, which argues that certain workplace stressors exhaust employees’ resources; therefore, their attitudes and behaviors toward completing their tasks are affected. Moreover, we also rely on the COR theory to explain the moderating role of IWE in the relationship between organizational cronyism and job outcomes, constructed on the belief that religious assets serve as a coping mechanism for employees which averts them from demonstrating negative attitudes and behaviors even on the face of organizational cronyism. We propose that it should not be supposed that all employees who are facing organizational cronyism, their work engagement will decrease in all situations. In line with the COR theory (Hobfoll, 2001), it is argued that some contextual factors can buffer the relationship between workplace stressors and workplace outcomes. By keeping in view the attribute of IWE, we choose IWE as the contextual mechanism in the relationship between workplace stressors and workplace outcomes. The practices of IWE help us in understanding that the relationship between organizational cronyism and work engagement could vary in the presence of IWE. The reactions of employees toward organizational cronyism would vary with high and low IWE beliefs. Therefore, we choose IWE as a contextual factor that could buffer the relationship between organizational cronyism and work engagement.

### Organizational Cronyism and Employee Performance

According to the available literature, organizational cronyism is considered a workplace stressor; therefore, it harms employees’ positive attitudes and behaviors such as job satisfaction, organizational commitment, and organizational citizenship behavior. Therefore, we assume a negative impact of organizational cronyism on employee performance. There are several reasons why organizational cronyism is negatively associated with employee performance (Khatri, 2017). Such as organizational cronyism is based on personal relationships rather than objective measures (Khatri and Tsang, 2003; Khatri et al., 2006); therefore, a vibrant concept of in-group members (e.g., cronies) and out-group members (e.g., non-cronies) prevails in organizations where organizational cronyism exists (Khatri and Tsang, 2003). Cronies get privilege in the appraisal, reward allocation, and other organizational affairs, but non-cronies are discriminated in all the stated aspects (Khatri and Tsang, 2003; Arasli and Tumer, 2008). Due to the reason, employees who encounter discrimination, they respond with impaired job satisfaction, commitment, and trust in the manager as well as in the organizations (Keles et al., 2011; Turan, 2015; Iqbal, 2016; Saleem et al., 2018; Yasmeen et al., 2019). All these negative factors have an eventual adverse impact on employee’s performance (Yang and Hu, 2009; Saleem et al., 2018). In-group members are highly trusted, supported, and rewarded; therefore, they enjoy high-performance ratings from their leaders due to having in-group status (Leung et al., 2008). However, the other side of the picture is worse, for instance, out-group members are disrespected, distrusted, and less supported by their leader due to having out-group tags (Akca, 2019). Therefore, out-group members’ motivation to perform their duties dwindles due to having an unjust reward allocation and recognition system (Khatri and Tsang, 2003; Imran et al., 2019).

Employees’ perception of their job is influenced by their manager’s behavior, which has an ultimate positive or negative impact on employees’ attitudes toward their job and performance (Akuffo and Kivipõld, 2019). For instance, when employees feel that they have not been treated on merit, they feel vexed, dissatisfied, and frustrated which ultimately hurt their energies to perform their job in a better way (Baris Erdem, 2015). Organizational cronyism has several undesirable work outcomes such as job dissatisfaction, deviant workplace behavior, low organizational commitment, and employee disengagement with their work (Turhan, 2014). The perception of organizational cronyism entails unfair, partial, and inequitable treatment with others, due to the reason it is considered as a workplace stressor (Shaheen et al., 2017). In line with the COR theory (Hobfoll and Lilly, 1993), organizational cronyism (workplace stressor) may be a cause of reducing employee’s performance. Organizational cronyism as a workplace stressor can consume employee’s resources and energies to work. In this vein, researchers agree that employees need energy, stamina, and physical and mental attachment to complete a job accurately. However, the unavailability of all the stated aspects leads to poor performance. Among all other factors, managers’ unfair and subjective treatment harms employees’ energies to perform. Consistent with the COR theory (Hobfoll and Lilly, 1993), organizational cronyism as a workplace stressor reduces employees’ resources which has an eventual impact on their performance.

Employees’ good or bad performance is directly linked to the manager’s behavior (Skakon et al., 2010). Manager’s actions could increase or decrease employee performance; manager’s fair and equitable treatment with the subordinates enhances trust in leader, engagement with the work, and commitment with the organization (Adams and Freedman, 1976). While unfair and biased behavior of the leader increases job dissatisfaction, employee negligence and deviance behavior (Rafferty and Restubog, 2011; Javed et al., 2019). When employees perceived that their inputs are not equal to their outputs, they remained less satisfied, less engaged and less enthusiastic toward achieving their goals (Adams and Freedman, 1976). Consequently, their performance decreases. Therefore, based on the stated literature and theory, it is hypothesized:

***H1.*** *There is a negative relationship between organizational cronyism and employee performance.*

### The Mediating Role of Employee Work Engagement in the Relationship Between Organizational Cronyism and Employee Performance

Engagement is defined as an active psychosomatic condition (Parker and Griffin, 2011). Work engagement is defined as a positive psychological state that compels employees to devote themselves actively to their job and organization (Bailey et al., 2017). This definition is derived from the work of Khan (1990), who is known as a pioneer in investigating the concept of engagement in the workplace. Work engagement is behavioral, emotional, and cognitive attachment to the organization, whereas a disengaged employee is unresponsive, uninterested, detached, and withdrawn from his/her work (Salanova et al., 2005). There are different types of engagement, e.g., engagement with the task, with the organization, and with the manager. Engagement with the task is defined as “an employee exerting physical, emotional and mental energy in completing allocated task” (Rich et al., 2010). Engagement with the organization is defined as “the extent to which an employee is physically and psychologically involved in completing particular organizational assignment” (Saks, 2006). Engagement with the leader is defined as “employees who invest their physical, mental and emotional resources in achieving their employer’s success” (Barrick et al., 2015).

Work engagement helps employees in successfully managing stress-related work environments (Shimazu et al., 2018). An extensive literature demonstrates a positive relationship between employee work engagement and desirable job outcomes, e.g., job satisfaction, organizational commitment, and employee performance (Özer et al., 2017; Joplin et al., 2019). In current literature, several antecedents of work engagement such as leadership support, supervisory support, leader-member exchange, as well as, positive outcomes of work engagement have been identified, e.g., organizational commitment, organizational citizenship behavior, task performance and organizational performance (Rurkkhum and Bartlett, 2012; Lai et al., 2020; Tisu et al., 2020). Henceforth, factors that play their part in reducing employees’ work engagement have been considered by researchers and practitioners (Crawford et al., 2010). Due to this reason, we target the particular factor which has the capacity to reduce employees’ work engagement, known as organizational cronyism. When employees are fairly treated in all manners they are more engaged with their work and exhibit above-average performance (Joplin et al., 2019; Lai et al., 2020), but when they perceive that they are not treated on objective measures they remain less engaged with their duties; and as a result their performance decreases (Moliner et al., 2008; Yin, 2018). In other words, when employees feel that their manager and organization are impartial in decision making and are concerned about their well-being, they are more motivated and engaged with their duties and fulfill their tasks zealously (Lai et al., 2020). Hence, organizational cronyism is an integral antecedent of employee work engagement which has an ultimate effect on employee performance.

Equity theory (Adams and Freedman, 1976) and the COR theory (Hobfoll and Lilly, 1993) provide sound theoretical support to explain why employees prefer to remain more or less engaged with their work as well as with the organization. Equity theory argues that employees’ reactions are dependent upon the manager’s actions; if employees are not treated equitably, they show less engagement and withdraw their energies to perform. The perception of employees about the unfair and inequitable treatment due to organizational cronyism influences employee engagement which, in return, affects their performance. Therefore, we predict the mediating role of work engagement in the relationship between organizational cronyism and employee performance. Following the COR theory (Hobfoll and Lilly, 1993), we suggest that the indirect effect of organizational cronyism on employee performance occurs through employee engagement. Therefore, it is hypothesized:

***H2.*** *Employee work engagement mediates the relationship between organizational cronyism and employee performance.*

### Moderating Role of Islamic Work Ethics in the Relationship Between Organizational Cronyism and Employee Performance

There are several contextual factors that can affect the relationship between organizational cronyism and individual’s reactions toward injustice and organizational cronyism (Khatri, 2011; Turhan, 2014; Saleem et al., 2018). For example, the role of leadership styles and cultural settings can possibly affect the individual’s job outcomes. Leadership style can influence the perception of injustice as a leader is responsible for the allocation of punishment and rewards to his followers. However, sometimes this allocation is not in an equitable manner (Javed et al., 2019). Therefore, researchers emphasize the investigation of those leadership styles which have the potential to influence the decisions of allocation of rewards and punishments to followers. The destructive leader behavior results in reduced employee well-being, deviant workplace behavior, lower organizational commitment, satisfaction and performance (Xu and Thomas, 2011; Baysak and Yener, 2015; Asrar-ul-Haq and Kuchinke, 2016). Accordingly, abusive supervision is negatively associated with follower’s belief in justice (Rafferty and Restubog, 2011). Sušanj and Jakopec (2012) found that different leadership styles such as passive leadership and active leadership style, do influence the organizational commitment and justice perception (Strom et al., 2014).

Leaders also play a direct or indirect role in shaping employee’s engagement (Popli and Rizvi, 2016). Leaders can boost employee engagement by providing on factors such as supportive environment, teamwork, inputs in decision making, fair treatment, and justice in the allocation of resources (Kahn, 1990; Kompaso and Sridevi, 2010; Jacobs, 2013; Barrick et al., 2015). Strom et al. (2014) suggested that transformational and transactional leadership styles can moderate the relationship between organizational justice and work engagement. Similarly, Saks (2006) found that employee engagement is influenced by supervisor’s support, perceived organizational support, procedural and distributive justice as well as employee’s personal resources such as stamina, self-efficacy, and optimism which result in employee performance.

Similarly, employees’ response toward injustice is subject to cultural orientation (Liu et al., 2013). Further, different cultural orientations also influence employee’s attitudes and behaviors. For example, employees belonging to high power distance cultures experience more injustice and favoritism in contrast to those belonging to low power distance cultures (Clugston et al., 2000). Employees belonging to high power distance cultures accept differences in decisions, allocation of resources by their leaders, and are less irritated by the directional nature of their leaders. Therefore, it is suggested that high power distance leads toward satisfactoriness of social discrimination (Liu et al., 2013). Similarly, employees belonging to low power distance cultural orientation are more concerned with justice as compared to employees belonging to high power distance cultural orientation (Mueller and Wynn, 2000).

A manager’s behavior is not the same toward all their subordinates (Scandura, 1999). Some are treated with extraordinary favor, while others could be discriminated in different situations (Javed et al., 2019). Therefore, employee’s adverse response (such as deviant workplace behavior, low organizational commitment, intention to quit the job, and job dissatisfaction) against organizational cronyism is quite logical and rational (Turhan, 2014). However, the researchers agreed that certain contextual factors could lessen the negative impact of organizational cronyism on employee outcomes (Khatri and Tsang, 2003). Therefore, we focus on IWE as a moderating mechanism in the relationship between organizational cronyism and employee outcomes. We suggest that employees with high IWE choose to remain engaged and perform better in all circumstances. We support this argument with the help of COR theory (Hobfoll and Lilly, 1993) and justify IWE as a coping mechanism which Muslim workers use to maintain their energies in unfavorable circumstances.

IWE refers to the orientation of the believer that shapes and affects his/her contribution and involvement in the workplace. IWE entails that work is a virtue by taking into consideration individual’s needs, and is essential to strike a balance between one’s personal and social life. It leads to life fulfillment and rejects life denial by embracing business purpose in the utmost regard. It leads to the expansion of one’s self-interest monetarily, socially and psychologically. IWE help to maintain social status, improve society’s welfare, and reiterate faith (Ali and Al-Owaihan, 2008). According to organizational cronyism literature, employees who face organizational cronyism at the workplace, they react with negative workplace attitudes and behaviors (Shaheen et al., 2017). IWE also teaches Muslim workers to fight against injustice and discriminatory system. But due to having a high power distance culture and controlled system every employee could not fight against the system. Therefore, we highlight the importance of IWE as a coping mechanism that motivates the Muslim workers to remain engaged with their work and perform better even in injustice circumstances. We argue that employees with high IWE do not get disappointed and depressed but they remain satisfied and conform to the unfavorable condition by utilizing their coping resources such as IWE.

Employees with high IWE pay less attention to the unfair treatment of managers and remain engaged and involved in their duties (Javed et al., 2019). This is due to the attribute of IWE, which motivates employees to avoid all types of undesirable attitudes and behaviors in the workplace (Khan et al., 2013; Alam and Talib, 2015). Therefore, when employees face organizational cronyism, the practices of IWE provide them with feelings of optimism and support (Khan et al., 2013; Murtaza et al., 2016). As a result, instead of responding with negative behaviors, non-cronies encounter the situation patiently and courageously and try to deal with undesirable situations.

An extensive literature on organizational cronyism suggests that when employees experience unfair treatment in the workplace, their perception of fairness is breached; therefore, they seem less interested, less committed, and less satisfied in the workplace (Arasli and Tumer, 2008; Karakose, 2014; Gupta et al., 2016; Saleem et al., 2018). However, all these negative behaviors are prohibited in Islam because Islam deters employees from negative behaviors and, in its place, strongly encourages commitment and hard work (Alam and Talib, 2015). Teachings of Qur’an and Hadith of Prophet Muhammad (Peace Be upon Him) encourage hard work and discourage idleness. For instance, it is stated in Qur’an, “there is not for a man except that for which he strives. O you who have believed, obey Allah, and obey the messenger, and do not void your deeds” (Qur’an, 53:39; 47:33). The phrase “do not void your deeds” means do not waste your time by engaging in futile activities. From an Islamic viewpoint, hard work means consistency and engagement in one’s work, which means employees who adhere to the practices of IWE remain engaged with their work and ultimately achieve the desired results sooner or later depending upon the will of Allah Almighty (Ali and Al-Owaihan, 2008). Allah’s Prophet said, “Do good deeds properly, sincerely, and moderately and know that your deeds will not make you enter Paradise and that the most beloved deed to Allah is the most regular and constant even if it were little” (Bukhari, 472). The saying of Prophet depicts that consistency and engagement are the key components to one’s success. When an employee observes organizational cronyism in various activities such as in the allocation of rewards, evaluation of assignments, and training and development opportunities, it decreases employee engagement, which harms their performance (Turhan, 2014). Though, employees with high IWE are less likely to respond to the biased treatment of their managers (Ali and Al-Owaihan, 2008).

According to IWE, employee’s job assignments are considered as ethical obligations that are to be fulfilled even in the absence of a transparent system (Bouma et al., 2003; Ali and Al-Owaihan, 2008). Believers are answerable for their acts to Allah Almighty; therefore, they have to follow those pathways which are acceptable by Allah and His Prophet (Peace Be Upon Him) in Islam. Due to this fact, IWE motivates employees to perform good deeds and avoid wrong deeds (Murtaza et al., 2016). In Islam, hard work is considered as a source of pleasure and achievement, and a true Muslim is defined as who completes his/her work with involvement, struggle and a high level of determination (Abu-Saad, 2003). For example, Prophet (Peace Be Upon Him) taught the Muslims that hard work is not only a source of sins to be remitted but the best food is one which has been earned with full devotion and effort (Ali and Al-Owaihan, 2008). He (Peace Be Upon Him) also defined the work as worship. Henceforth, these examples motivate employees to remain engaged with their work rather than disengagement with the work. In addition, employees practicing IWE are highly committed to perform their duties with transparency, integrity and energy which motivates them to achieve the desired results with persistent determination (Yousef, 2001; Ali and Al-Owaihan, 2008).

Moreover, Islam strongly discourages dishonesty, laziness, and shirking of work in all phases of business operations. Since organizational cronyism directs employees toward a breach of fairness perception which ultimately decreases their engagement with their assigned duties but employees with high IWE remain engaged and committed with their work due to the unique attribute of IWE. Likewise, research on religiosity recommends religion as a critical driver of the individual’s belief system and it significantly impacts the intrinsic and extrinsic work values of the individuals (Ali et al., 1995). Under the umbrella of the teachings of the Qur’an and Sunnah [teachings of Prophet Muhammad (Peace Be Upon Him)], we suggest that IWE moderates the relationship between organizational cronyism and work engagement.

The COR theory (Hobfoll and Lilly, 1993) suggests that certain coping mechanisms exist, which employees use to protect their resources in stressful situations. Organizational cronyism acts as a workplace stressor for employees, and as a consequence, due to the depletion of resources, they remain less engaged with their work. However, an employee with high IWE focuses on his/her duties with integrity and devotion without paying attention to the practices of organizational cronyism. Therefore, we considered IWE as a religious motivator and valuable resource for employees, which they use to maintain and manage stressful situations at the workplace. Researchers suggest that employees having high IWE and low IWE respond differently to stressful situations. For instance, Javed et al. (2019) concluded that employees having high IWE demonstrated less deviant workplace behavior in the presence of abusive supervision. Also, Khan et al. (2013) suggested that employees with high and low IWE behave differently toward job outcomes. In line with the COR theory (Hobfoll and Lilly, 1993), we suggest IWE as a religious motivator which offers employees with valuable resources such as belief, hope, optimism, positive affect and life satisfaction which motivate them to remain engaged and sincere with their duties even in unfavorable circumstances such as organizational cronyism.

By using the conceptualization of COR theory and drawing on the literature of IWE, we hypothesize:

***H3.*** *Islamic work ethics moderates the relationship between organizational cronyism and employee work engagement such that this relationship will be stronger when Islamic work ethics is high.*

**FIGURE 1:**
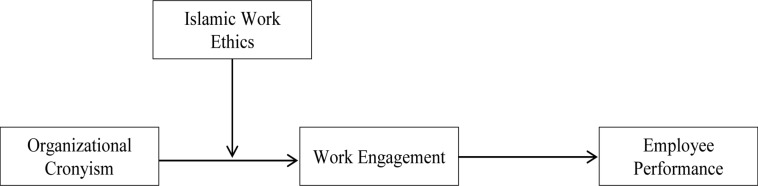
Conceptual framework.

The conceptual framework is presented in Figure 1.

## Materials and Methods

### Sample and Data Collection Procedure

Data were collected from doctors, nurses, and healthcare staff with the help of self-administered questionnaires. Before the distribution of questionnaires, the principal author personally visited public sector hospitals located in different geographical locations of Punjab, Pakistan, and took permission from the heads of the hospitals. After getting permission, the questionnaires were distributed to doctors, nurses, and staff. The data were collected in two time waves. At each time wave, we attached a cover letter with the questionnaire comprising of detailed information about the purpose of the study and ensuring the anonymity of their responses. Moreover, it was also clarified that there is no right and wrong answer to any question and that their response is valuable for us. In the first wave, data were collected on the items related to organizational cronyism and IWE and work engagement. In the second wave, after 1 month, the data were collected on the items related to employee performance. Since the purpose of the time-lag design is to overcome the common method bias and differentiate the independent variable from the dependent variable; due to that reason, we collected data in T1 (time wave 1) regarding independent variable (organizational cronyism), moderating variable (IWE) and mediating variable (employee engagement), and in T2 (time wave 2) data were collected regarding dependent variable (employee performance).

Responses on employee performance were taken from the heads and supervisors of the departments of the hospitals. For instance, if we collected the data from eight healthcare staff members working in the department of cardiology, the head of the cardiology department filled the questionnaire on employee performance of those eight healthcare workers. Both questionnaires were properly coded so that both responses could be matched for the purpose of data analysis. Of the circulated 320 questionnaires, 267 properly filled and completed responses were used for data analysis having a response rate of 83.43%. The demographics demonstrated that 62.9% were male, and 37.1% were females; 59.6% were married and 40.04% were unmarried. Concerning employee experience, 6% had five or less than 5 years’ experience; 14.6% had 6–10 years of experience; 50.02% had 11–15 years of experience, and 29.02% had 16 or more than 16 years of experience. Related to professional status, nurses were 10.9% of the total sample, house officers were 17.2% and postgraduate residents were 71.9% of the total respondents. Moreover, 13.09, 7.5, 42.3, 36.03% fell under the age category of 20–25, 26–30, 31–35, 36 and above, respectively.

### Measures

All the variables were measured on a 5 point Likert-type scale with anchors “1 = Strongly Disagree, 2 = Disagree, 3 = Neutral, 4 = Agree, 5 = Strongly Agree.” Among four theoretical variables, three variables organizational cronyism, IWE, and employee engagement were self-reported, while employee performance was assessed with the help of the supervisor’s ratings.

#### Organizational Cronyism

Organizational cronyism was measured by using the 7-items scale developed by Arasli and Tumer (2008). Examples of items contain “Politicians and political affinities are connected to being appointed, promoted, and the various decision-making activities of in this organization,” and “Sex discrimination at this organization damages the profitability and motivation of other employees.” This scale is further validated by Erdem and Karataş (2015) and Akuffo and Kivipõld (2019).

#### Work Engagement

Work engagement was measured by using a 6-items scale developed by Barrick et al. (2015). The sample items of the scale were “I really throw myself into my work” and “I devote a lot of effort and energy to my work.” This scale is further validated by Keles et al. (2011) and Joplin et al. (2019).

#### Islamic Work Ethics

IWE was measured by using the 17-items scale developed by Ali (1992). The sample items of the scale contain “Laziness is a vice,” and “Dedication to work is a virtue.” Previous studies have established a good internal consistency for using this scale (e.g., Khan et al., 2013; Murtaza et al., 2016; Javed et al., 2018, 2019).

#### Employee Performance

Employee performance was measured by using a supervisor-rated 7-items scale developed by Williams and Anderson (1991). The sample items were “Adequately complete assigned duties,” and “Fulfills responsibilities specified in the job description.” This scale is also validated by Joplin et al. (2019), Reb et al. (2019) and Tufail et al. (2019).

## Results

### Measurement Model

We confirmed the measurement model through confirmatory factor analysis (CFA) before testing our hypotheses. The results of the CFA confirmed the distinctiveness of all the variables as well as proved that the model is free from common method bias. Table 1 represents that the four-factor model fits the data well and offers more considerable perfections in fit indices as in contrast to three-factor and one-factor models: χ^2^/*df* = 1.645, RMSEA = 0.04, IFI = 0.94, TLI = 0.940, CFI = 0.94 (Hair et al., 2006).

**TABLE 1:** Comparison of alternative measurement models for main constructs.

**Model**	**χ^2^**	**df**	**Δχ^2^**	**Δ*df***	**RMSEA**	**CFI**	**SRMR**
4-factor model	1012.183^∗^	615	–	–	0.049	0.945	0.052
3-factor model^a^	2463.10*	626	1450.92	11	0.105	0.744	0.126
1-factor model^b^	4994.945^∗^	629	3982.76	14	0.162	0.391	0.190

^a^Combining work engagement and employee performance. ^b^Combining all items. *p < 0.001.

### Common Method Bias

Owing to the limitations of Harman’s one-factor test, following Podsakoff et al. (2012), we tested our measurement model with and without a common latent factor (CLF) in order to assess the extent to which common method bias (CMB) is a serious problem in the data. A CLF is a latent factor in a measurement model that has a direct relationship with all the observed variables (indicators). A separate measurement model was run, including a CLF having direct paths to all the observed variables. The variance of the CLF was fixed to 1 (Gaskin, 2012). This model reported good fit to the data [χ^2^(614) = 1007.880, p < 0.01; χ^2^/DF = 1.641; RMSEA = 0.049; SRMR = 0.0512; CFI = 0.945; TLI = 0.940].

To assess the extent of the CMB, the standardized factor loadings of the measurement model with CLF and measurement model without CLF were compared with each other. Typically, the standardized factor loadings of the measurement model without CLF would be higher than those in the measurement model with CLF because CMB tends to inflate the correlations. CMB could pose a potential threat if these differences are big, i.e., > 0.20 (Gaskin, 2012). In our data, these differences were trivial (ranging from 0.019 to 0.067), and no individual difference in standardized factor loadings of the model with and without CLF was greater than 0.20. Moreover, only 2% variance was explained by the common method factor, which is well below the threshold of 25% (Williams et al., 1989). These results show that CMB is not a problem and could be ruled out.

### Descriptive Statistics, Correlation Analysis, Average Variance Extracted (AVE), and Square Root of AVE

Table 2 represents descriptive statistics, means, standard deviations, correlation analysis, average variance extracted (AVE), and the square root of AVE for all the theoretical variables. The construct validity of the model was measured through convergent validity and discriminant validity (Hair et al., 2016). The AVE values proved the convergent validity of the proposed model. The values of AVE were higher than the threshold criteria, which is 0.5, as suggested by Hair et al. (2006) and Kelly and Pruitt (2015). Hence, these results verified the convergent validity of the model (see Table 2). The discriminant validity of all the variables was also established by using additional criteria suggested by Fornell and Larcker (1981), according to this criterion the square root of AVE of all the variables should be greater than their correlations. In our case, the square root of AVE of all the variables was greater than their correlations, as presented on diagonals in Table 2. Moreover, the Cronbach’s alpha (α) reliabilities of organizational cronyism, IWE, work engagement, and employee performance were 0.93, 0.94, 0.92, and 0.93, respectively, which are also meeting the threshold criteria which is 0.6 (Hair et al., 2014). According to the correlation results, organizational cronyism related negatively with IWE (*r* = -0.052, *P* > 0.05), employee work engagement (*r* = -0.320, *P* < 0.01), and with employee performance (*r* = -0.189, *P* < 0.01). IWE related positively to employee work engagement (*r* = 0.247, *P* < 0.01) and employee performance (*r* = 0.312, *P* < 0.01). Employee work engagement related positively to employee performance (*r* = 0.154, *P* < 0.05). Correlation analysis depicts that all associations are in the expected direction.

**TABLE 2:** Descriptive statistics, correlations, and discriminant validity.

	**Variables**	**Mean**	***SD***	**CR**	**α**	**AVE**	**1**	**2**	**3**	**4**	**5**	**6**	**7**	**8**
1	Professional status	2.05	0.77		–	–	–							
2	Experience	3.10	0.87		–	–	−0.09	–						
3	Age	2.87	0.86		–	–	0.00	−0.11	–					
4	Gender	1.24	0.42		–	–	−0.19**	−0.27**	0.05	–				
5	Organizational cronyism	2.98	0.90	0.92	0.93	0.66	0.031	0.14*	−0.13*	−0.039**	(0.81)			
6	Islamic work ethics	3.60	0.77	0.94	0.94	0.51	0–0.11	−0.18**	0.11	0.28**	−0.05	(0.71)		
7	Work engagement	4.00	0.78	0.93	0.92	0.69	0–0.07	−0.25**	0.10	0.26**	−0.32**	0.24**	(0.83)	
8	Employee performance	3.56	0.80	0.93	0.93	0.68	−0.07	−0.17**	0.13*	0.27**	−0.18**	0.31**	0.15*	(0.82)

N = 267, **p<0.01, *p<0.05. SD, Standard deviation; CR, Construct reliability; α, Cronbach’s alpha; AVE, Average variance extracted.

### Test of Hypotheses

**FIGURE 2:**
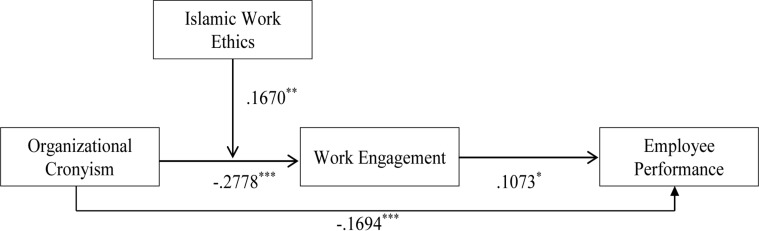
Results of the hypothesized model.

We tested the full hypothesized model by utilizing the method proposed by Preacher et al. (2007). Particularly, the hypotheses were analyzed using the PROCESS macro developed by Hayes (2013). We used PROCESS model 7 to test the moderated mediation analysis, which is fairly suitable for our hypothesized model. The results of the mediation analysis are presented in Table 3. According to hypothesis 1, organizational cronyism was negatively associated with employee performance. The statistical results were in line with the hypothesized relationship (β = −0.139, *p* < 0.05); thus, hypothesis 1 was accepted. According to Hypothesis 2, organizational cronyism was expected to have an indirect effect on employee performance through employee engagement. The indirect effect of organizational cronyism on employee performance was proved to be substantial as demonstrated by the 90% Bootstrapped confidence interval which did not include zero (-0.0580; -0.0046). The statistical results proved mediation in the direct relationship, thus, hypothesis 2 was accepted. The results for hypothesis 1 and hypothesis 2 are also presented as path values in Figure 2.

**TABLE 3:** Results of the mediation analyses (without covariates).

	**Coefficient**	**SE**	**Bootstrap 90% CI**
*IV to the mediator (a path)*			
Cronyism → Engagement	−0.2778***	0.0506	
*Mediator to DV (b path)*			
Engagement →Performance	0.1073*	0.0654	
*Total effect of IV on DV (c path)*	−0.1694***	0.054	
*Direct effect of IV on DV* (ć path)	−0.1396**	0.0568	
*Indirect effect of IV on DV through the proposed mediator*			
Cronyism → Engagement → Performance	−0.0298*	0.0162	[−0.0580; −0.0046]

*p < 0.10, **p < 0.05, ***p < 0.01.

**FIGURE 3:**
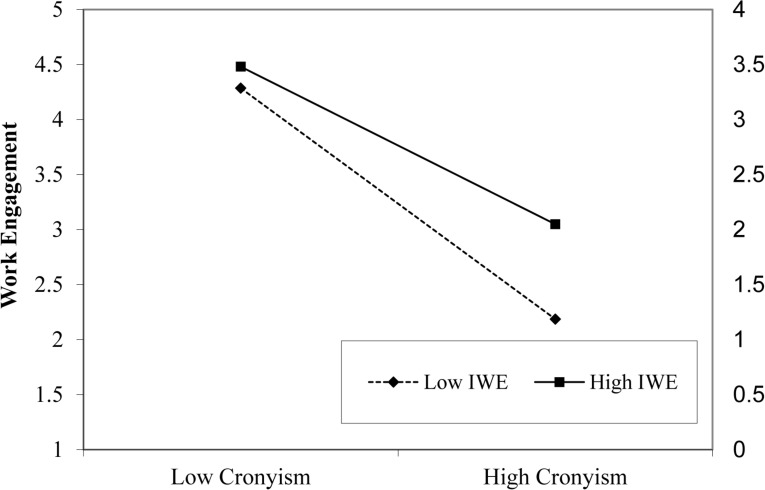
Moderating effect of Islamic work ethics on the relationship between organizational cronyism and work engagement.

The results of hypothesis 3 are exhibited in Table 4. The findings presented in Table 4 direct that IWE moderated the relationship between organizational cronyism and employee work engagement (β = 0.167, *p* < 0.05). According to our expectations, the association was stronger for employees who have high IWE. Thus, hypothesis 3 was accepted. Following the steps suggested by Aiken et al. (1991), we further plotted the significant interacting effect by computing slopes 1 SD above and below the mean of IWE (see Figure 3). This interaction shows that work engagement is high when cronyism is low coupled with high IWE.

**TABLE 4:** Ordinary least squares regression coefficients from moderated mediation model.

	**Outcome**			
**Predictors**	***M:*Engagement**		***Y:*Performance**	
Constant	5.6999		3.5503	
*X:*Cronyism	−0.8831***		−0.1396**	
*M:*Engagement			0.1073*	
IWE	−0.2349			
Cronyism × IWE	0.1670**			
*R*^2^	0.1718		0.0455	
F	18.1809***		6.2988***	
Moderator	Index of moderated mediation		95% Confidence Interval based on 5,000 bootstraps resamples	
Islamic work ethics	0.0179		−0.0017−0.0503	
**Conditional indirect effects of IWE = Mean ± 1SD**				

**IWE**	**Bootstrap Indirect Effect**	**Bootstrap SE**	**Boot LLCI**	**Boot ULCI**

2.8728	−0.0433	0.0252	−0.0988	0.0007
3.8200	−0.0263	0.0147	−0.0574	0.0004
4.2900	−0.0179	0.0112	−0.0426	0.0004

N = 267. PROCESS Model 7, Bootstrap sample size = 5,000; LL, lower limit; UL, upper limit; CI, confidence interval. *p < 0.10, **p < 0.05, ***p < 0.01 (two-tailed).

## Discussion and Conclusion

Drawing on the COR theory (Hobfoll and Lilly, 1993), we developed a framework assuming that work engagement mediates the negative relationship between organizational cronyism and employee performance. We also suggested that IWE moderates the relationship between organizational cronyism and work engagement. The statistical results of this time-lagged study were adjuvant for our proposed model. According to the results of the study, organizational cronyism (workplace stressor) was negatively associated with employee performance (employee behavior). Our results are consistent with the COR theory (Hobfoll and Lilly, 1993), which suggests that certain workplace stressors such as organizational cronyism exhaust employees’ resources; therefore, their performance is affected. This negative relationship is also supported by Turhan (2014), who suggests when non-cronies perceive that they are less supported, rewarded, and recognized as compared to the cronies, they respond with more destructive behaviors and less supportive behavior, therefore, their performance decreases. These results are also in line with several studies, such as Khatri and Tsang (2003).

As anticipated, work engagement mediated the relationship between organizational cronyism and employee performance. This is quite logical and supported by the COR theory (Hobfoll and Lilly, 1993) that unpleasant events such as organizational cronyism reduced work engagement; therefore, their performance decreased. This logical link is well supported by the literature and theory, but not investigated empirically, particularly, in the healthcare sector of Pakistan. Everyday print media and social media report several incidents about the poor performance of nurses and doctors in public sector hospitals of Pakistan. This urges the researchers to investigate the reasons and underlying mechanisms of their poor performance.

We suggest that among other reasons for doctors’ poor performance, organizational cronyism is one integral factor that is underemphasized in public sector hospitals of Pakistan. We not only investigate the causes of nurses’ and doctors’ poor performance but also investigate a complete mechanism through which their performance is affected. This novel investigation of the mediating role of employee engagement paints a true picture of the doctors’ poor performance. According to this link, when nurses and doctors experience unfairness, injustice in rewards allocation, and allocation of assignments, they respond with disengagement with their work, and due to this disengagement, their performance decreases. Moreover, IWE (contextual factor) moderates the relationship between organizational cronyism and employee performance, such as the relationship is stronger with high IWE. Thus, the COR theory (Hobfoll and Lilly, 1993), and available literature on organizational cronyism provide strong support for the findings of this study.

The outcomes of this study extend the organizational behavior literature in numerous ways. First, this study fills the gap in the literature by examining the role of practices of organizational cronyism in reducing employee performance with the lens of employee work engagement. By investigating the mediation link, we test a complete path on how organizational cronyism reduces employee performance. To the best of our knowledge, this is the first study that investigates the mediating role of work engagement in the relationship between organizational cronyism and employee performance in the healthcare sector of Pakistan. In line with the COR theory (Hobfoll and Lilly, 1993), doctors’ dedication, passion, and vigor with their work are affected when they perceive that they are not equally treated; therefore, they seem less interested in executing their job tasks accurately. Second, the examination of the moderating role of IWE provides new insights into the literature on organizational cronyism. The results of the study depicted that employees with high IWE fulfill their duties with sincerity and responsibility even while facing organizational cronyism. Due to the unique attribute of IWE, they remained engaged with their work. Therefore, their performance did not imperil. The investigation of the unique contextual findings enriches the current literature on organizational cronyism. We suggest that employees having strong Islamic values and belief systems remain less concerned with the injustice and impartial treatment of the management. Instead, they seem satisfied, calm, optimistic, and confident and fulfill their assigned duties as moral obligations; therefore, their performance is never jeopardized. Third, we chose the healthcare sector of Pakistan to investigate the proposed model. Which helps us in understanding the reason and underlying mechanism, why the performance of nurses and doctors is questionable in the healthcare sector of Pakistan? Fourth, the proposed model has been tested through the lens of the COR theory. By implementing the COR theory on the literature on organizational cronyism, the proposed model clearly explains the complete path how certain work events such as organizational cronyism reduces employee engagement which has an eventual impact on their performance; additionally, how does IWE motivate employees to remain engaged with their work even while facing discrimination.

## Theoretical Implications

This study entails several theoretical contributions. It uses the COR theory (Hobfoll and Lilly, 1993) to explain the impact of workplace stressor, organizational cronyism on detrimental work outcomes such as disengagement of employees and poor employee performance that indicates exhaustion of one’s resources. We used IWE as a form of personal resources that could be used to cope with the depletion of resources. Thus, we have tried to extend the scope of personal resources in the COR theory. The findings of the study also suggest IWE as a coping mechanism that helps the employees in extenuating the effect of organizational cronyism, which assists them to remain engaged with their work and perform better (Hobfoll and Lilly, 1993). This study suggests that Islamic values and beliefs play their role as religious motivators that compel employees to avoid all types of misdeeds and endorse ethical behaviors at the workplace. Islamic beliefs and values reinforce honesty, hard work, and punctuality at the workplace and avoid all types of wrongdoings (Ali and Al-Kazemi, 2007). The findings of this study suggest that employees’ moral values grounded on religiosity can influence employees’ workplace attitudes and behaviors. By adding IWE as a contextual factor in the literature on organizational cronyism, we provide new insight into the literature. The existing literature on organizational cronyism suggests that the practices of organizational cronyism reduce positive employee attitudes and behaviors and increase negative employee attitudes and behaviors (Khatri and Tsang, 2003; Turhan, 2014; Saleem et al., 2018). But several cultural and contextual factors can alter this relationship. Therefore, we introduce IWE as a contextual factor in the relationship between organizational cronyism and employee attitudes and behaviors. To the best of our knowledge, this is the first study to investigate IWE as a contextual contributor to the relationship between organizational cronyism and employee’ work outcomes. By doing so, we also tried to fill the gaps in the literature mentioned by Khatri and Tsang (2003) and Turhan (2014). The application of COR theory (Hobfoll and Lilly, 1993) is also helpful in understanding the generalizability of this theory in eastern culture.

## Practical Implications

The findings of the study have several implications for the management of the healthcare sector of Pakistan. First, the performance of the doctors is dependent upon the treatment of the leaders and managers. To improve the performance of the doctors, they must be treated in a just manner. Management and leaders should adopt a transparent, equitable, and impartial system of promotion, resource allocation, and performance management for the healthcare personnel. Second, it is concluded in this study that IWE plays a role in minimizing negative behaviors and fostering positive behaviors in the workplace. Hence, Islamic values and beliefs have an impact on employees’ ethical behaviors. It is found in our study that the employees who have high IWE pay less attention to organizational cronyism and remain engaged with their work; they, therefore, perform better in contrast to those employees who have low IWE. It means management and leaders should focus on the principles of IWE and should work on promoting IWE. Third, IWE could be promoted at the workplace through pieces of training and seminars, which are needed to be arranged not only at employees’ level but also at the managerial level. So that organizational cronyism could be minimized, and an impartial working environment could be promoted. In this way, the adverse reaction toward organizational cronyism could be lessened, as well as; it is also a moral way to improve the performance of the employees.

## Limitations and Future Research Directions

Despite having numerous contributions, this study also has some limitations. First, we select only public sector hospitals for data collection. However, a clear picture could be presented if a comparison could be performed by collecting data both from the public as well as private sector hospitals. Future studies can make such comparisons to extend the findings on this research. Second, our sample size was relatively small; we believe the generalizability of the results could be better with an increased sample size. Third, the data were collected only from public sector hospitals of Pakistan which could limit the external validity of this study. Hence, future researchers can conduct this study in different Muslim countries and further incorporate cultural context to widen the scope of this study. In the current study, we examined IWE as a coping mechanism that can motivate employees to be engaged and perform better in all circumstances. But it will be interesting for the future researchers to examine the retaliatory actions of employees toward organizational cronyism and see how strong IWE help fight against injustice and how the holders of this strong morality respond to injustice.

## Data Availability Statement

The raw data supporting the conclusions of this article will be made available by the authors, without undue reservation, to any qualified researcher.

## Ethics Statement

The studies involving human participants were reviewed and approved by ethics committee of Government College University Faisalabad, Pakistan. The patients/participants provided their written informed consent to participate in this study.

## Author Contributions

SSh, SZ, and SSa: definition of the research objectives, models, hypotheses, principal manuscript crafting, and data analysis plan and data analysis. SSh, SSa, and GS: the provision of materials (i.e., questionnaires). SSh, and GS: data collection. SZ ad SSa: manuscript revision and proofreading. SSh, SZ, SSa, and GS: final approval. All authors contributed to the article and approved the submitted version.

## Conflict of Interest

The authors declare that the research was conducted in the absence of any commercial or financial relationships that could be construed as a potential conflict of interest.

## References

[B1] Abu-Saad I. (2003). The work values of arab teachers in Israel in a multicultural context. *J. Beliefs Values* 24 39–51. 10.1080/1361767032000052980

[B2] Adams J. S. Freedman S. (1976). Equity theory revisited: comments and annotated bibliography. *Adv. Exp. Soc. Psychol.* 9 43–90. 10.1016/S0065-2601(08)60058-1

[B3] Aiken L. S. West S. G. Reno R. R. (1991). *Multiple Regression: Testing and Interpreting Interactions.* Thousand Oaks, CA: Sage.

[B4] Akca M. (2019). “An investigation of the relationship between favoritism and workplace deviance: meditation role of negative emotions,” in *Analyzing Workplace Deviance in Modern Organizations*, ed. Sharma N. (Hershey, PA: IGI Global), 114–138. 10.4018/978-1-5225-9996-8.ch007

[B5] Akuffo I. N. Kivipõld K. (2019). Influence of leaders’ authentic competences on nepotism-favouritism and cronyism. *Manag. Res. Rev.* 43 369–386. 10.1108/MRR-02-2019-0078

[B6] Alam M. A. Talib N. (2015). Islamic work ethics and individualism in managing a globalized workplace: does religiosity and nationality matter? *J. Manag. Organ.* 1 1–17. 10.1017/jmo.2015.54

[B7] Ali A. J. Al-Kazemi A. A. (2007). Islamic work ethic in Kuwait. *Cross Cult. Manag.* 14 93–104. 10.1108/13527600710745714

[B8] Ali A. J. Al-Owaihan A. (2008). Islamic work ethic: a critical review. *Cross Cult. Manag.* 15 5–19. 10.1108/13527600810848791

[B9] Ali A. J. (1992). The Islamic work ethic in Arabia. *J. Psychol.* 126 507–519. 10.1080/00223980.1992.10543384

[B10] Ali A. J. Falcone T. Azim A. A. (1995). Work ethic in the USA and Canada. *J. Manag. Dev.* 14 26–34. 10.1108/02621719510086156

[B11] Ali A. J. Gibbs M. (1998). Foundation of business ethics in contemporary religious thought: the ten commandment perspective. *Int. J. Soc. Econ.* 25 1552–1564. 10.1108/03068299810214089

[B12] Arasli H. Tumer M. (2008). Nepotism, favoritism and cronyism: a study of their effects on job stress and job satisfaction in the banking industry of North Cyprus. *Soc. Behav. Personal.* 36 1237–1250. 10.2224/sbp.2008.36.9.1237

[B13] Aryee S. Budhwar P. S. Chen Z. X. (2002). Trust as a mediator of the relationship between organizational justice and work outcomes: test of a social exchange model. *J. Organ. Behav.* 23 267–285. 10.1002/job.138

[B14] Asrar-ul-Haq M. Kuchinke K. P. (2016). Impact of leadership styles on employees’ attitude towards their leader and performance: empirical evidence from Pakistani banks. *Future Bus. J.* 2 54–64. 10.1016/j.fbj.2016.05.002

[B15] Bailey C. Madden A. Alfes K. Fletcher L. (2017). The meaning, antecedents and outcomes of employee engagement: a narrative Synthesis. *Int. J. Manag. Rev.* 19 31–53. 10.1111/ijmr.12077

[B16] Baris Erdem A. K. (2015). The effects of cronyism on job satisfaction and intention to quit the job in hotel enterprises: the case of three, four and five star hotels in Muðla, Turkey. *MANAS Sosyal Araştırmalar Dergisi* 4 55–74.

[B17] Barrick M. R. Thurgood G. R. Smith T. A. Courtright S. H. (2015). Collective organizational engagement: linking motivational antecedents, strategic implementation, and firm performance. *Acad. Manag. J.* 58 111–135. 10.5465/amj.2013.0227

[B18] Baysak B. Yener M. Ý (2015). The relationship between perceived leadership style and perceived stress on hospital employees. *Proc. Soc. Behav. Sci.* 207 79–89. 10.1016/j.sbspro.2015.10.159

[B19] Beekun R. I. (2019). Islamic BUSINESS ETHIcs. *Islamic Bus. Ethics* 20170 1–80. 10.2307/j.ctvk8w1zv.4

[B20] Begley T. M. Khatri N. Tsang E. W. K. (2010). Networks and cronyism: a social exchange analysis. *Asia Pacific J. Manag.* 27 281–297. 10.1007/s10490-009-9137-4

[B21] Bernstein M. J. Sacco D. F. Young S. G. Hugenberg K. Cook E. (2010). Being “in” with the in-crowd: the effects of social exclusion and inclusion are enhanced by the perceived essentialism of ingroups and outgroups. *Personal. Soc. Psychol. Bull.* 36 999–1009. 10.1177/0146167210376059 20693384

[B22] Bouma G. Haidar A. Nyland C. Smith W. (2003). Work. religious diversity and Islam. *Asia Pacific J. Hum. Resour.* 41 51–61. 10.1177/1038411103041001022

[B23] Büte M. (2011). The effects of nepotism and favoritism on employee behaviors and human resources practices: a research on Turkish public banks. *TODAÝE’s Rev. Public Adm.* 5 185–208.

[B24] Clugston M. Howell J. P. Dorfman P. W. (2000). Does cultural socialization predict multiple bases and foci of commitment? *J. Manag.* 26 5–30. 10.1177/014920630002600106

[B25] Crawford E. R. LePine J. A. Rich B. L. (2010). Linking job demands and resources to employee engagement and burnout: a theoretical extension and meta-analytic test. *J. Appl. Psychol.* 95 834–848. 10.1037/a0019364 20836586

[B26] Daðli A. Akyol Z. (2019). The relationship between favouritism behaviours of secondary school administrators and organizational commitment of the teachers. *J. Educ. Train. Stud.* 7 35–49. 10.11114/jets.v7i7.4191

[B27] Eastman W. Santoro M. (2003). The importance of value diversity in corporate life. *Bus. Ethics Q.* 13 433–452. 10.5840/beq200313431

[B28] Erdem B. Karataş A. (2015). The effects of cronyism on job satisfaction and intention to quit the job in hotel enterprises: the case of three, four and five star hotels in Muðla. Turkey. *Manas J. Soc. Stud.* 4 54–74.

[B29] Fornell C. Larcker D. F. (1981). Evaluating structural equation models with unobservable variables and measurement error. *J. Market. Res.* 18 39–50. 10.2307/3151312

[B30] Gaskin J. (2012). Common method bias using common latent factor. *Gaskination’s Statistics.* Available online at: http://youtube.com/Gaskination (accessed January 22, 2012).

[B31] Gupta V. Agarwal U. A. Khatri N. (2016). The relationships between perceived organizational support, affective commitment, psychological contract breach, organizational citizenship behaviour and work engagement. *J. Adv. Nurs.* 72 2806–2817. 10.1111/jan.13043 27293180

[B32] Hair F. J. Jr. Hult G. T. M. Ringle C. Sarstedt M. (2016). *A Primer on Partial Least Squares Structural Equation Modeling (PLS-SEM).* Thousand Oaks, CA: Sage International.

[B33] Hair J. F. Black W. C. Babin B. J. Anderson R. E. Tatham R. L. (2006). Multivariate data analysis 6th Edition. *J. Abnorm. Psychol.* 49 103–104. 10.1198/tech.2007.s455 12611515

[B34] Hair J. F. Sarstedt M. Hopkins L. Kuppelwieser V. G. (2014). Partial least squares structural equation modeling (PLS-SEM): an emerging tool in business research. *Eur. Bus. Rev.* 26 106–121. 10.1108/EBR-10-2013-0128

[B35] Hayes A. (2013). *Introduction to Mediation, Moderation, and Conditional Process Analysis (Methodology in the Social Sciences).* New York, NY: The Guilford Press.

[B36] Hobfoll S. E. (2001). The influence of culture, community, and the nested-self in the stress process: advancing conservation of resources theory. *Appl. Psychol.* 50 337–370. 10.1111/1464-0597.00062

[B37] Hobfoll S. E. Lilly R. S. (1993). Resource conservation as a strategy for community psychology. *J. Commun. Psychol.* 21 128–148. 10.1002/1520-6629(199304)21:2<128::AID-JCOP2290210206<3.0.CO;2-5

[B38] Imran M. K. Iqbal J. Fatima T. Iqbal S. M. J. Jamal W. N. Nawaz M. S. (2019). Why do i contribute to organizational learning when i am ostracized? A moderated mediation analysis. *J. Manag. Organ.* 21 128–148. 10.1017/jmo.2019.70

[B39] Iqbal Q. (2016). Preferential treatment: an empirical study in education sector of pakistan. *Int. J. Manag. Account. Econ.* 3 486–497.

[B40] Jacobs H. (2013). *An Examination of Psychological Meaningfulness, Safety, and Availability as the Underlying Mechanisms linking Job Features and Personal Characteristics to Work Engagement.* Dissertation, Florida International University, Miami, FL, 10.25148/etd.FI13080518.

[B41] Javed B. Fatima T. Yasin R. M. Jahanzeb S. Rawwas M. Y. A. (2019). Impact of abusive supervision on deviant work behavior: the role of Islamic work ethic. *Bus. Ethics* 28 221–233. 10.1111/beer.12212

[B42] Javed B. Rawwas M. Y. A. Khandai S. Shahid K. Tayyeb H. H. (2018). Ethical leadership, trust in leader and creativity: the mediated mechanism and an interacting effect. *J. Manag. Organ.* 24 388–405. 10.1017/jmo.2017.56

[B43] Johnson T. Grim B. Najmul P. Hashmi I. Bharti S. Maurya N. K. (2013). The World’s religions in figures: an introduction to worldwide religious demography. *Soc. Change* 22 34–37. 10.1177/0049085715618558

[B44] Joplin T. Greenbaum R. L. Wallace J. C. Edwards B. D. (2019). Employee entitlement, engagement, and performance: the moderating effect of ethical leadership. *J. Bus. Ethics* 10.1007/s10551-019-04246-0

[B45] Kahn W. A. (1990). Psychological conditions of personal engagement and disengagement at work. *Acad. Manag. J.* 33 692–724. 10.5465/256287

[B46] Karakose T. (2014). The effects of nepotism, cronyism and political favoritism on the doctors working in public hospitals. *Stud. Ethno Med.* 8 245–250. 10.1080/09735070.2014.11917640

[B47] Keles H. N. Ozkan T. K. Bezirci M. (2011). A study on the effects of nepotism, favoritism and cronyism on organizational trust in the auditing process in family businesses in turkey. *Int. Bus. Econ. Res. J.* 10 9–16. 10.19030/iber.v10i9.5622

[B48] Kelly B. Pruitt S. (2015). The three-pass regression filter: a new approach to forecasting using many predictors. *J. Econ*. 186 294–316. 10.1016/j.jeconom.2015.02.011

[B49] Khan K. Abbas M. Gul A. Raja U. (2013). Organizational justice and job outcomes: moderating role of islamic work Ethic. *J. Bus. Ethics* 126 235–246. 10.1007/s10551-013-1937-2

[B50] Khatri N. (2011). A taxonomy of supervisor-subordinate exchanges across cultures. *IIMB Manag. Rev.* 23 71–80. 10.1016/j.iimb.2011.03.002

[B51] Khatri N. (2017). “Definitions of cronyism, corruption, and crony capitalism,” in *Crony Capitalism in India*, eds Khatri N. Ojha A. K. (Basingstoke: Palgrave Macmillan), 3–7. 10.1007/978-1-137-58287-4_1

[B52] Khatri N. Tsang E. W. K. (2003). Antecedents and consequences of cronyism in organizations. *J. Bus. Ethics* 43 289–303. 10.1023/A:1023081629529

[B53] Khatri N. Tsang E. W. K. Begley T. M. (2006). Cronyism: a cross-cultural analysis. *J. Int. Bus. Stud.* 37 61–75. 10.1057/palgrave.jibs.8400171

[B54] Kompaso S. M. Sridevi M. S. (2010). Employee engagement: the key to improving performance. *Int. J. Bus. Manag.* 5 89–96. 10.5539/ijbm.v5n12p89

[B55] Lai F. Y. Tang H. C. Lu S. C. Lee Y. C. Lin C. C. (2020). Transformational leadership and job performance: the mediating role of work engagement. *SAGE Open* 10 1–11. 10.1177/2158244019899085

[B56] Leung T. K. P. Heung V. C. S. Wong Y. H. (2008). Cronyism: one possible consequence of guanxi for an insider: how to obtain and maintain it? *Eur. J. Market.* 42 23–34. 10.1108/03090560810840899

[B57] Liu C. Yang L. Q. Nauta M. M. (2013). Examining the mediating effect of supervisor conflict on procedural injustice-job strain relations: the function of power distance. *J. Occup. Psychol.* 18 64–74. 10.1037/a0030889 23276193

[B58] Marri M. Y. K. (2012). Measuring Islamic work ethics and its consequences on organizational. *Int. J. Manag. Sci. Bus. Res.* 2 37–49.

[B59] Moliner C. Martinez-Tur V. Ramos J. Peiro J. M. Cropanzano R. (2008). Organizational justice and extrarole customer service: the mediating role of well-being at work. *Eur. J. Work Organ. Psychol.* 17 327–348. 10.1080/13594320701743616

[B60] Mueller C. W. Wynn T. (2000). The degree to which justice is valued in the workplace. *Soc. Justice Res.* 13 1–24. 10.1023/A:1007515618127

[B61] Murtaza G. Abbas M. Raja U. Roques O. Khalid A. Mushtaq R. (2016). Impact of Islamic work ethics on organizational citizenship behaviors and knowledge-sharing behaviors. *J. Bus. Ethics* 133 325–333. 10.1007/s10551-014-2396-0

[B62] Özer Ö Uðurluoðlu Ö Saygili M. (2017). Effect of organizational justice on work engagement in healthcare sector of Turkey. *J. Health Manag.* 19 73–83. 10.1177/0972063416682562

[B63] Parker S. K. Griffin M. A. (2011). Understanding active psychological states: embedding engagement in a wider nomological net and closer attention to performance. *Eur. J. Work Organ. Psychol.* 20 60–67. 10.1080/1359432X.2010.532869

[B64] Peng J. C. Jien J. J. Lin J. (2016). Antecedents and consequences of psychological contract breach. *J. Manag. Psychol.* 31 1312–1326. 10.1108/JMP-10-2015-0383

[B65] Podsakoff P. M. MacKenzie S. B. Podsakoff N. P. (2012). Sources of method bias in social science research and recommendations on how to control it. *Annu. Rev. Psychol.* 63 539–569. 10.1146/annurev-psych-120710-100452 21838546

[B66] Popli S. Rizvi I. A. (2016). Drivers of employee engagement: the role of leadership style. *Glob. Bus. Rev.* 17 965–979. 10.1177/0972150916645701

[B67] Preacher K. J. Rucker D. D. Hayes A. F. (2007). Taylor & francis online?:: addressing moderated mediation hypotheses: theory. *Methods Prescript. Multi. Behav. Res.* 42 185–227. 10.1080/00273170701341316 26821081

[B68] Rafferty A. E. Restubog S. L. D. (2011). The influence of abusive supervisors on followers’ organizational citizenship behaviours: the hidden costs of abusive supervision. *Br. J. Manag.* 22 270–285. 10.1111/j.1467-8551.2010.00732.x

[B69] Reb J. Chaturvedi S. Narayanan J. Kudesia R. S. (2019). Leader mindfulness and employee performance: a sequential mediation model of LMX quality. Interpersonal Justice, and Employee Stress. *J. Bus. Ethics* 160 745–763. 10.1007/s10551-018-3927-x

[B70] Rich B. L. Lepine J. A. Crawford E. R. (2010). Job engagement: antecedents and effects on job performance. *Acad. Manag. J.* 53 617–635. 10.5465/amj.2010.51468988

[B71] Rurkkhum S. Bartlett K. R. (2012). The relationship between employee engagement and organizational citizenship behaviour in Thailand. *Hum. Resour. Dev. Int.* 15 157–174. 10.1080/13678868.2012.664693

[B72] Saks A. M. (2006). Antecedents and consequences of employee engagement. *J. Manag. Psychol.* 21 600–619. 10.1108/02683940610690169

[B73] Salanova M. Agut S. Peiró J. M. (2005). Linking organizational resources and work engagement to employee performance and customer loyalty: the mediation of service climate. *J. Appl. Psychol.* 90 1217–1227. 10.1037/0021-9010.90.6.1217 16316275

[B74] Saleem M. A. Yaseen A. Zahra S. (2018). Predictors of organizational commitment in public sector hospitals of pakistan—a moderated mediation study. *J. Health Manag.* 20 206–225. 10.1177/0972063418763656

[B75] Scandura T. A. (1999). Rethinking leader-member exchange: an organizational justice perspective. *Leadersh. Q.* 10 25–40. 10.1016/S1048-9843(99)80007-1

[B76] Shaheen S. Bashir S. Khan A. K. (2017). Examining organizational cronyism as an antecedent of workplace deviance in public sector organizations. *Public Pers. Manag.* 46 308–323. 10.1177/0091026017716655

[B77] Shaw A. Joseph S. Linley P. A. (2005). Religion, spirituality, and posttraumatic growth: a systematic review. *Ment. Health Relig. Cult.* 8 1–11. 10.1080/1367467032000157981

[B78] Shimazu A. Schaufeli W. B. Kubota K. Watanabe K. Kawakami N. (2018). Is too much work engagement detrimental? Linear or curvilinear effects on mental health and job performance. *PLoS One* 13:e0208684. 10.1371/journal.pone.0208684 30586369 PMC6306155

[B79] Skakon J. Nielsen K. Borg V. Guzman J. (2010). Are leaders’ well-being, behaviours and style associated with the affective well-being of their employees? A systematic review of three decades of research. *Work Stress* 24 107–139. 10.1080/02678373.2010.495262

[B80] Strom D. L. Sears K. L. Kelly K. M. (2014). Work engagement: the roles of organizational justice and leadership style in predicting engagement among employees. *J. Leadersh. Organ. Stud.* 21 71–82. 10.1177/1548051813485437

[B81] Sušanj Z. Jakopec A. (2012). Fairness perceptions and job satisfaction as mediators of the relationship between leadership style and organizational commitment. *Psihol. Teme* 21 509–526.

[B82] Tisu L. Lup?a D. Vîrgã D. Rusu A. (2020). Personality characteristics, job performance and mental health the mediating role of work engagement. *Personal. Individ. Differ.* 153:109644 10.1016/j.paid.2019.109644

[B83] Tsui A. S. Nifadkar S. Ou A. Y. (2007). Cross-national, cross-cultural organizational behavior research: advances, gaps, and recommendations. *J. Manag.* 33 426–478. 10.1177/0149206307300818

[B84] Tufail M. Sultan F. Anum (2019). Examining the effect of challenge-hindrance stressors on work attitude and behavior. *FWU J. Soc. Sci.* 13 90–104.

[B85] Turan A. (2015). Does the perception of organizational cronyism leads to career satisfaction or frustration with work? The mitigating role of organizational commitment. *Rese. Appl. Econ.* 7:14 10.5296/rae.v7i3.8164

[B86] Turhan M. (2014). Organizational cronyism: a scale development and validation from the perspective of teachers. *J. Bus. Ethics* 123 295–308. 10.1007/s10551-013-1839-3

[B87] van Gilst E. Schalk R. Kluijtmans T. Poell R. (2020). The role of remediation in mitigating the negative consequences of psychological contract breach: a qualitative study in the banking sector. *J. Change Manag.* 20 264–282. 10.1080/14697017.2020.1737180

[B88] Williams L. J. Cote J. A. Buckley M. R. (1989). Lack of method variance in self-reported affect and perceptions at work: reality or artifact? *J. Appl. Psychol.* 74 462–468. 10.1037/0021-9010.74.3.462

[B89] Williams L. J. Anderson S. E. (1991). Job statisfiction and organizational commitment as predictors of organizational citizenship behaviors. *J. Manag.* 17 601–617.

[B90] Xu J. Thomas H. C. (2011). How can leaders achieve high employee engagement. *Leaders. Organ. Dev. J.*. 32 399–416. 10.1108/01437731111134661

[B91] Yang Y. Hu B. (2009). “RETRACTED ARTICLE: the antecedents of organizational cronyism,” in *Proceedings - International Conference on Management and Service Science, MASS 2009*, Wuhan, 1–4. 10.1109/ICMSS.2009.5304810

[B92] Yasmeen R. Bibi M. Raza A. (2019). Impact of organization politics on human resource management practices and employee performance. *SEISENSE J. Manag.* 2 39–47. 10.33215/sjom.v2i2.118

[B93] Yin N. (2018). The influencing outcomes of job engagement: an interpretation from the social exchange theory. *Int. J. Product. Perform. Manag.* 67 873–889. 10.1108/IJPPM-03-2017-0054

[B94] Yousef D. A. (2001). Personnel review. *Disabil. Employ.* 30 152–169. 10.1108/00483481011075611

